# Professional development short scale: Measurement invariance, stability, and validity in Brazil and Angola

**DOI:** 10.3389/fpsyg.2022.841768

**Published:** 2022-07-22

**Authors:** Luciana Mourão, Susana M. Tavares, Hugo Sandall

**Affiliations:** ^1^Programa de Pós-Graduação em Psicologia, Universidade Salgado de Oliveira, Niterói, Brazil; ^2^Programa de Pós-Graduação em Psicologia Social, Universidade do Estado do Rio de Janeiro, Rio de Janeiro, Brazil; ^3^Business Research Unit (BRU-IUL), Instituto Universitário de Lisboa (ISCTE-IUL), Lisboa, Portugal

**Keywords:** professional development, assessment, psychometric properties, invariance, self-efficacy, in-role performance, job satisfaction, career promotion

## Abstract

Professional development is the vital process in the workplace that comprises the growth and maturation of knowledge, skills, and attitudes arising from formal and informal learning at work throughout one’s life. The goal of this research was to present validity evidence and accuracy of the Professional Development Short Scale (PDSS) for different occupational categories. The research was conducted using four cross-sectional questionnaire surveys with convenience samples of different occupational categories (*N* = 2,547) in 41 cities throughout Brazil and Angola. The first study aimed to explore the factorial structure and internal consistency of the PDSS. The second study aimed to evaluate the cross-cultural validity and measurement invariance of the scale. The third study was to assess concurrent validity and predictive validity. The fourth study was to assess the test–retest reliability. The results indicated a one-factor structure, with six items for both countries’ datasets. This research pointed out the validity of the PDSS as regards its convergence-discriminant pattern with the General Self-Efficacy and Job Self-Efficacy Scales, and also, the relationship of the PDSS with relevant constructs (Bases of Power/leadership styles, In-role performance, Job Satisfaction, and Career Promotion). In this study, we provide psychometric validity of the Professional Development Short Scale to offer it as a resource to measure the construct and allow researchers to apply it in research models easily integrated to other constructs. We covered several different incremental approaches to ensure the scale validity. Besides showing temporal stability to ensure it can be applied from time to time, as one dynamic construct should, we also indicated that social desirability did not influence the measurement of the PDSS. Furthermore, the results indicate that the effects of the method do not generate undue confusion on the scale. Thus, the psychometric properties of the PDSS allow for recommending the use of the scale in extensive studies. This scale therefore contributes to contemporary professional development literature through the comparison of the perceptions of professional development in different professional categories and by providing organizational researchers with a tool to evaluate the effects and predictors of such construct.

## Introduction

Professional development is the growth and maturation of knowledge, skills, and attitudes arising from formal and informal learning at work throughout one’s life (Mourão and Fernandes, 2020). Therefore, professional development is a natural consequence of training actions (Bell et al., 2017) but depends not only on training (Dachner et al., 2021). It is linked to any kind of learning focused on the workplace (Fernandes et al., 2019).

As theoretical background, we consider the trans-occupational model of professional development proposed by Fernandes et al. (2019), with five dimensions: Work Context, Motivation, Training/Learning, Relational Elements, and Lived Experiences. According to these authors, Professional Development goes beyond the normative definitions of a profession. It involves an individual learning process, which takes place over time, involving knowledge, skills, and attitudes used in work situations. In this sense, the development of professional skills would be a process that occurs throughout one’s working life.

Research on employee development pointed to behavioral (being involved in development) and situational (support for the development of employees in an organization) variables, in addition to stable individual difference variables as predictors (Maurer and Chapman, 2013). Organizations should develop their human resources from a broader learning perspective, including continuous learning, informal learning, and knowledge sharing (Noe et al., 2014). Despite this, Continuing Professional Development (CPD) is a field of study that is new and undefined, even in the case of teachers, who are an occupational category with more research on this topic (Hill et al., 2013).

From a multidisciplinary and multilevel perspective, training and development activities can benefit individuals, teams, organizations, and society (Aguinis and Kraiger, 2009; Bell et al., 2017). In addition, professional development has been characterized as a social demand and has been associated with autonomy, motivation, job performance, and job satisfaction (Shah et al., 2011; Borg, 2018). Furthermore, there is a trend toward a growing demand for personal and professional development on the part of both employees and those applying for jobs (Cascio, 2019).

Despite being a recent issue, this topic has been studied in different fields such as Building Information Modelling (Bosch-Sijtsema et al., 2019), Accounting (Paisey and Paisey, 2020), Education (Bates and Morgan, 2018; Borg, 2018), Health (Allen et al., 2019), and Psychology (Dawson, 2018; Chernaya and Obukhova, 2019).

Moreover, professional development derives from a set of opportunities and practices, such as (a) training processes (Noe et al., 2014; Bell et al., 2017; Fernandes et al., 2019); (b) supervision (getting guidance and monitoring the actions of the student or new professionals); (c) integrated strategies (formal and informal learning for competence development; Mourão and Fernandes, 2020); (d) in-service training (Shah et al., 2011); (e) participation in communities of practice (Patton and Parker, 2017); (f) experiential learning based on critical actions and reflections of the workers about their professional daily life (Fernandes et al., 2019); and (g) interaction of the workers with their labor context (Bell et al., 2017).

The area of the literature review suggests that there are two main categories of professional development predictors: training and development activities (formal learning) and experiential learning (informal learning; Mourão and Fernandes, 2020). The first category is receiving greater attention from organizations (Aguinis and Kraiger, 2009). The second category has a greater impact in the learning process focused on the work of the formal actions taken in courses or educational institutions (Illeris, 2011; Mourão and Fernandes, 2020). This category finds support in the Experiential Learning Theory (Kolb, 2014), a holistic perspective that simultaneously considers experience, perception, cognition, and behavior (Fernandes et al., 2019). According to Kolb (2014), “learning is the process whereby knowledge is created through the transformation of experience” (p. 49). Kolb’s theory considers a cyclical model of learning, based on four steps, namely, concrete experience, reflective observation, abstract conceptualization, and active experimentation. The experimental learning cycle is therefore associated with the verbs to do, to observe, to think, and to plan and shows how experience is translated through reflection into concepts.

The learning stages presented by Kolb (2014) show that the process of professional development necessarily involves a maturity that comes with time. So, for this study, professional development is a process that results in different types of learning on the job, being related to professional skills acquired over time, and allows workers to improve their work performance and do their job satisfactorily. In this sense, the items of the professional development scale, according to the theoretical concept, should reflect the temporal issue.

In the same direction, professional development comprises a series of events and activities occurring over time that creates an outcome where those involved have gained various skill sets and current knowledge in their area of expertise (Mourão and Fernandes, 2020). The concept of professional development focuses on processes involving a set of modes of learning, formal and informal, having cognitive as well as behavioral and affective dimensions. Professional development is directly activated by daily life activities and should be part of a broader process of continuous learning (Bates and Morgan, 2018; Mourão and Fernandes, 2020).

Professional development appears in the literature to be linked to leadership. Namely, professional development has been associated with transformational leadership (Mourão, 2018). Transformational leaders view their team’s intellectual stimulation as critical, as it encourages a high level of performance from analyzing and exploring their practice. Therefore, professional development can be induced by leaders. This proposition finds support in the Learning at Work Model from Illeris (2011), which has comprised two processes: social, which is external, understood as an interaction between the individual and their social environment and cultural materials, and the psychological, which is internal, understood as acquisition and development where new impulses are connected with the results of prior learning.

Although the concept of professional development is commonly used to designate induced learning activities, a broader conceptualization views professional development as a result of various types of learning at work, whether through formal or informal actions (Mourão and Fernandes, 2020). Informal learning actions at work relate to searching for new knowledge and skills, usually considering any demand or need to be associated with individuals’ tasks. It happens outside the curricula of educational programs from planned or unplanned experiences (Fernandes et al., 2019; Mourão and Fernandes, 2020) and may occur from a doubt remedied by a more experienced colleague, by reading books and documents of the organization, through daily observation, workplace learning, etc. Thus, the workplace can be considered a place of continuous knowledge production through an informal learning process that takes place in parallel with the actions of the training system.

Many studies assess learning in the workplace (Aguinis and Kraiger, 2009; Kwon et al., 2020), but instruments that measure the degree of professional development for workers are missing in the literature. Furthermore, most research on the topic is qualitative, or measures the professional development specifically for a particular profession or career, using specific indicators for that profession (e.g., Bates and Morgan, 2018; Allen et al., 2019; Bosch-Sijtsema et al., 2019; Paisey and Paisey, 2020), but Fernandes et al. (2019) pointed out similarities in the processes of professional development in different careers (Engineering, Medicine, Psychology, and Law).

Although there are studies dealing with different professions and occupations, the literature in the area indicates that research on professional development still predominantly adopts qualitative methodologies. Standardized scales allow comparisons and advances in quantitative studies. Reliable and valid measurement is critical for advancing research and evidence-based work practice. Thus, the new scale is vital to enable the advancement of studies on the subject. Therefore, this paper aims to present evidence of the validity and accuracy of Short Scale Professional Development that can be used in all organizational and occupational settings.

In Psychology, there has been a growing interest in the development of short scales, including for trait measurements, which are typically long scales (Romero et al., 2012). Short scales have also been used in healthcare to monitor population prevalence and trends (Kessler et al., 2002). As in other fields, in organizational research, short scales are needed because surveys with many variables tend to increase fatigue, boredom, and burden for participants, which can affect data quality. Schweizer (2011) confirms the trend of using short scales. He analyzed the European Journal of Psychological Assessment issues published in 2010 and pointed out several published scales with only five or even four items. According to the author, another advantage is that short scales tend to have a higher degree of homogeneity than those composed of a minimum of 10–12 items. Furthermore, according to congeneric test theory, short scales are more likely to endure an investigation by means of confirmatory factor analysis (Lucke, 2005).

Particularly in the case of research on professional development, scales used are longer and specific to each professional category. This limitation makes it difficult for the construct to be investigated more widely. It also prevents the comparison of results between different professional categories, which hinders the performance of the human resources manager. Hence, it is essential to have measures to assess professional development in different situations with different audiences and collect data while including other variables.

On the above, a short scale of professional development may contribute to the contemporary professional development literature by permitting the comparison of the perceptions of professional development in different professional categories and by providing a tool for researchers to assess the antecedent and consequent variables of this construct. Professional development from a small scale can be advantageous because there is a set of variables that can be consistent with professional development, such as job performance, improving products and services, and customers’ welfare. A scale of a few items can allow models to combine more antecedents and consequences in research on the topic. In addition, the scale may be helpful to assess the results of specific learning in work practices as well as to measure the employees’ professional development over time.

To meet this goal, we explored the factor structure, internal consistency, criterion validity, and measurement invariance in cross-cultural validity (Brazil and Angola) of the Professional Development Short Scale (PDSS). We also evaluated predictive validity (job satisfaction and contextual performance), criterion validity (career promotion), and temporal stability of the scale.

## Present research

This research was conducted using four cross-sectional questionnaire surveys with convenience samples of different occupational categories in 41 cities across Brazil and Angola. Both countries are examples of collectivistic cultures from two different continents. This aspect highlights the individual’s loyalty toward the social groups with which they identify, beyond their immediate family, which can impact the individual’s professional development. Thus, because many scales are designed and test evidence of validity in individualistic contexts, our purpose was to conduct such a process originally in collectivist countries (Triandis, 2018).

The study was conducted with the application of printed questionnaires, and online submission was allowed, when participants opted for this type of submission. The study was previously approved by a research ethics committee in Brazil and we complied with all precepts expected from a study with human beings. Such precepts encompassed the confidentiality of personal information, the right to voluntary participation, and to quit the research, in accordance with the Declaration of Helsinki.

The first study aimed to explore the factor structure, internal consistency, and criterion validity of the PDSS. The second study aimed to evaluate the cross-cultural validity and measurement invariance of the scale. The third sought to assess content validity and criterion validity. Concerning the increased emphasis on the validity of the PDSS tests, it is essential to remember that validity was considered as a pivotal property of a scale (Schweizer, 2011). Finally, the fourth study was to assess the test–retest reliability. To achieve the multiple testing goals of the scale, we performed successive data collections, as shown in Table 1.

**Table 1:** Analysis procedures adopted and respective samples.

Purpose	Sample 1 (*N* = 251^b^)	Sample 2 (*N* = 546^b^; *N* = 626^a^)	Sample 3 (*N* = 179^b^)		Sample 4 (*N* = 240^b^)
			Time 1 (*N* = 203)	Time 2 (*N* = 179)	
Confirmatory factor analysis	X				
Cross-cultural invariance		X			
Temporal stability			X		
Method effect^1^			X		
Convergent validity^2^				X	X
Discriminant validity^3^					X
Criterion validity^4^		X		X	

a Angola.

b Brazil.

1 Method effect (Social Desirability; Job satisfaction).

2 Convergent validity (Leadership Based on Expert Power) and divergent validity (Leadership Based on Reward Power).

3 Discriminant Validity (General Self-Efficacy and Job Self-Efficacy Scales).

4 Criterion validity (In-Role Performance; Career Promotion).

### Study 1: Confirmatory factor analysis

Study 1 aimed for the evaluation of the latent model of the PDSS structure. To that end, we performed the confirmatory factor analysis (CFA). The first version of the scale was developed by Mourão et al. (2014). As such, we chose to carry out the CFA directly because there is clearly a theoretical framework for the PDSS. Confirmatory analysis is a significantly more rigorous test of the measurement model and latent structure of professional development.

#### Materials and methods

##### Participants

Participants in various studies were recruited through one of two means (email or in person). All prospective participants joined voluntarily and were informed about the general aim of the respective study and were invited to complete a questionnaire containing demographic questions, the Professional Development Short Scale and other scales were used for validity testing. The research was predominantly applied face-to-face. Some participants (less than 10%) requested that the questionnaire be sent by email. In this case, they scanned the printed version of the completed questionnaire and sent it to the researchers. In this sense, there was no online application of the survey, and the only difference was the return of the printed or scanned questionnaire. No participants were paid or received any direct benefit for participating in these studies. The sample for the CFA analyses involved 251 workers (41.0% were female). The age of the sample ranged from 19 to 64 (*M* = 33.67, *SD* = 9.68). Regarding education, 53.4% had completed college. Working time ranged from 1 to 40 years (*M* = 13.30, *SD* = 9.66).

##### Measures

###### Professional development short scale

This measure was constructed by Mourão et al. (2014) to assess the current state of professional development of workers in different occupations. The theoretical definition of the construct served as the basis for defining properties of the construct that the built items should address. The wording of items was drawn from the analysis of the discursive body raised by conducting in-depth interviews with 10 workers (five women and five men) from different public and private sectors (trade, health, education, public safety organizations, and the manufacturing industry). The authors performed an analysis of judges which included seven experts who evaluated the relevance of the items to measure the construct. The one-factor scale was designed to measure professional development, assessed by eight items on a seven-point response scale: 1 (*strongly disagree*) to 7 (*strongly agree*).

#### Results

The data were analyzed in JASP statistical software, version 0.13 (JASP Team, 2020). The CFA was performed using the Weighted Least Squares Means and Variances (WLSMV) estimation method, considering the ordinal nature of the items. The eight items, their factor loadings, and commonalities are provided in Table 2. It gained good internal consistency with a 0.82 Cronbach’s *α* and McDonald’s Ω coefficient.

**Table 2:** Communalities and loading factors.

Items	Communalities	Loading factors (CFA)
1. I have everything necessary for the completion of my work skills.	0.36	0.79
2. My boss has already made compliments about my development as a professional.	0.21	0.74
3. I have had a significant professional development since I started working.	0.50	0.77
4. I think that my performance has improved as a professional.^*^	0.40	0.63
5. My colleagues rave about my professional growth.^*^	0.26	0.66
6. With my current knowledge, I can do my job satisfactorily.	0.49	0.81
7. I have become a more qualified professional.	0.43	0.76
8. Currently, I feel well prepared to undertake activities that are meant for me.	0.47	0.83

* excluded; Cronbach’s *α* = 0.82; McDonald’s Ω = 0.82.

Considering the intention to have a short version of the scale (Kessler et al., 2002; Lucke, 2005; Schweizer, 2011; Romero et al., 2012), three criteria were established for the maintenance of the items, specifically: (a) factor loading (cutoff of 0.70, indicating 49% overlapping variance, according to Hair et al., 2019 recommendations), (b) items that added aspects of professional development that are not covered by other items, and (c) an adequate level of reliability considering the set of items kept in the scale. Thus, six items from the original scale were kept for the CFA.

The analysis of the items that reached the loading factors of 0.70 cutoff shows that items that included the temporal question remained in the PDSS, whether speaking of it directly (“I have had significant professional development since I started working” and “I have become a more qualified professional”); whether addressing the present moment with the idea that there was a change over time (“Currently, I feel well prepared to undertake activities that are meant for me” and “With my current knowledge, I can do my job satisfactorily”). This temporal element that characterizes the items that reached the highest factor loading is in line with the Professional Development Trans-occupational Model proposed by Fernandes et al. (2019). According to this model, professional development involves an individual process of improving skills that takes place over time. From the learning and opportunities arising from the Work Context, Motivation, Training/Learning, Relational Elements, and Lived Experiences, workers develop throughout their working life.

There are also items that refer to the achievement of a certain stage of professional development, either from the person’s own perception (“I have everything necessary for the completion of my work skills”) or from the boss’s praise (“My boss has already made compliments about my development as a professional”). The first concerns the reflective dimension of professional development, while the second concerns feedback from bosses; both items are theoretically supported by elements considered crucial in the professional development process (Mourão, 2018).

The other two items, in addition to not reaching the factor loading of 0.70, had other reasons for being excluded from the scale. One of them (“I think that my performance has improved as a professional”) contemplates the temporal dimension that is already present in the four previously mentioned items. In this sense, no additional contribution was identified that would justify maintaining this item. The other item removed from the scale concerns the peer assessment (“My colleagues rave about my professional growth”), which may represent a less reliable indicator of professional development, since some workers do not work in work teams. Finally, maintaining these two items did not improve the reliability of the scale, since both the eight-item version and the six-item version had Cronbach’s *α* = 0.82 and McDonald’s Ω = 0.82.

The model fit evaluation was based on sample size independent indices such as the Root-Mean-Square Error of Approximation (RMSEA), Standardized Root Mean Residual (SRMR), the Tucker–Lewis index (TLI), and the Comparative Fit Index (CFI), as well as the χ^2^ test statistic and an evaluation of parameter estimates (χ^2^ = 29.75, *df* = 20.00, *p* = 0.07). The PDSS-CFA solution provides a good fit to the data (CFI = 0.992, TLI = 0.989, RMSEA = 0.004, and SRMR = 0.006). Either values greater than 0.90 on the CFI and TLI, and RMSEA values lower than 0.06, or values greater than 0.95 on the CFI and TLI, and RMSEA values lower than 0.08 typically reflect acceptable and excellent fits to the data (Gana and Broc, 2018).

### Study 2: Cross-cultural validity

The second survey was to evaluate the cross-cultural validity and measurement invariance. Therefore, the main aim of the present study was to simultaneously contribute to the validation of the Brazilian (BR) and Angolan (AO) versions of the Professional Development Short Scale through a cross-cultural investigation, aimed at (1) replicating the validation of the Brazilian scale upon new data and (2) comparing both versions of the PDSS (BR and AO) by exploring their measurement equivalence (Vandenberg and Lance, 2000; Cheung, 2008).

Brazil and Angola were chosen for this study for two reasons: both countries speak Portuguese and were experiencing high growth rates at the time of data collection. Furthermore, as the construct studied was workers’ professional development, the fact that the countries were growing rapidly was an essential shared aspect. Specifically, we hypothesize that the BR version of the PDSS will show the same one-factor structure found in the validation presented in Study 1. Furthermore, we hypothesize that the AO version of the PDSS should show at least configural equivalence (Cheung, 2008). This would allow testing of other, more rigorous forms of equivalence.

**Figure 1:**
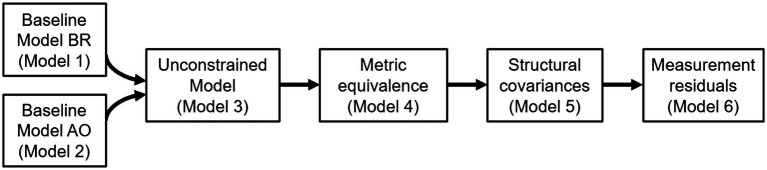
Flow diagram of the cross-cultural validity and measurement equivalence.

We ran a series of multiple-group CFA, through which we tested different forms of equivalence (Vandenberg and Lance, 2000; Cheung, 2008). In addition to configural equivalence (i.e., the number of observed variables associated with each construct would be the same across groups), the following forms of equivalence may be tested: metric equivalence, or equivalence in factor loadings, structural covariance (loadings factor + covariances equals), and measurement residuals (loadings factor + covariances + residuals equals). Tests of configural equivalence and metric equivalence were the first two steps. Structural covariance could have been a third step; however, this form of equivalence was not required for testing the remaining forms of equivalence (Cheung, 2008). Finally, we also tested the Measurements residuals for the BR and AO datasets (Figure 1). The following criteria were adopted to confirm the invariance: CFI (>0.90), TLI (>0.90), RMSEA (<0.08; Gana and Broc, 2018), and ΔCFI (<0.01; Putnick and Bornstein, 2016).

#### Materials and methods

##### Participants

The sample of the second study contained 546 Brazilian (58.1% male) and 626 Angolan workers (59.6% male) of multiple occupations. The age ranged from 18 to 64 (*M* = 33.36, *SD* = 9.64) and 19 to 65 (*M* = 33.89, *SD* = 8.37), respectively, to Brazil and Angola. Concerning education, 50.9% had completed college in the Brazilian sample and 42.3% in the Angolan sample.

##### Measures

###### Professional development short scale

The one-factor scale is used to measure PDSS, each assessed by six items on a seven-point response scale: 1 (*strongly disagree*) to 7 (*strongly agree*), according to the CFA results in Study 1. The internal consistency indices were satisfactory. In the Brazilian sample, the value obtained for Cronbach’s α and McDonald’s Ω coefficient was 0.79. In the Angolan sample, the value obtained was slightly higher, both the Cronbach’s α coefficient and the McDonald’s Ω coefficient were 0.84.

#### Results

This section reports the results for the Multiple-Group CFA. Table 2 reports the results of a series of CFA carried out on the PDSS data. Preliminary analyses conducted separately on the BR and AO datasets confirmed that the one-factor solution of the PDSS was a good fit in both BR (Table 2, Model 1) and the AO (Table 2, Model 2). Thus, Model 1 and Model 2, which configured a one-factor solution of the PDSS, were taken as the baseline models for the following sequence of multiple group analyses through which measurement equivalence was tested (Figure 1).

The first multiple-group analysis tested an unconstrained model (Table 3, Model 3) by simultaneously evaluating the fit of Model 1 and Model 2. The practical fit indices (SRMR = 0.044, RMSEA = 0.049, CFI = 0.992) indicated a good fit for this model, supporting an equivalent one-factor solution of the PDSS both in the BR dataset and the AO dataset. Model 4 tested for metric equivalence (factor loadings). While the χ^2^ of Model 4 deteriorated compared to that of Model 3 [Δχ^2^_M4-M3(3)_ = 9.26], all the fit indices of Model 4 were acceptable. More importantly, a ΔCFI_M3-M4_ = −0.001 suggested that Model 4 could be considered equivalent to Model 3. Thus, measurement weights were also supported. By applying the same logic, we supported structural covariance as tested by Model 5 [Δχ^2^_M5-M4(1)_ = 19.726, ΔCFI_M4-M5_ = −0.005]. Finally, Model 6, testing for measurement residuals (see Figure 1) was nested within Model 4. However, constraining item intercepts to be equal across samples determined a drastic deterioration in the χ^2^ [Δχ^2^_M6-M5(6)_ = 29.787], a ΔCFI_M4-M6_ = −0.008 suggested that measurement residuals were not supported (Table 3).

**Table 3:** The goodness of fit statistics for tests of cross-cultural equivalence of the PDSS in Brazil and Angola.

	*χ* ^2^	*df*	SRMR	RMSEA	TLI	RMSEA (90%)	CFI	·CFI
Model 1	28.746	9	0.052	0.063	0.977	0.04–0.09	0.986	—
Model 2	14.626	9	0.037	0.032	0.995	0.00–0.06	0.997	—
Model 3	43.373	18	0.044	0.049	0.987	0.03–0.07	0.992	−0.005
Model 4	52.629	23	0.049	0.047	0.988	0.03–0.06	0.991	−0.001
Model 5	72.355	28	0.050	0.052	0.985	0.04–0.07	0.986	−0.005
Model 6	102.142	34	0.064	0.059	0.981	0.05–0.07	0.978	−0.008

Model 1: 1-factor, Brazil, *N* = 546; Model 2: 1-factor, Angola, *N* = 626; Model 3: multiple-group, unconstrained equivalence; Model 4: multiple-group, metric equivalence; Model 5: multiple-group, structural covariances equivalence; and Model 6: multiple-group, measurement residuals equivalence.

The series of multiple-group CFA presented in Table 3 demonstrated that we tested different forms of equivalence (Vandenberg and Lance, 2000; Cheung, 2008). We confirmed the measurement invariance. These results supported our hypotheses that the factor structure would fit the BR dataset and that the same solution would be equivalent to the AO in terms of the number of factors, the factor loadings of the items, the values of random intercepts, and the residuals of the items.

### Study 3: Convergent, discriminant, criterion validity, and method effects

The third study aims to advance the validation of the PDSS by exploring its correlations with criterion-related variables. This study tested Convergent, Discriminant, and Criterion Validity of the Professional Development Short Scale. Furthermore, we considered that social desirability could produce response bias in professional development perception. Therefore, we included the Social Desirability Scale—SDS (Stöber, 2001) in CFA to test Method Effects (Paulhus, 1991). Convergent validity and discriminant validity, respectively, reflect the extent to which a measure relates to similar constructs and does not relate to constructs from which it should differ.

In this section, hypotheses are formulated concerning the constructs to which professional development should (General Self-Efficacy and Job Self-Efficacy Scales, Leadership Based on Expert Power) and should not be related (Leadership Based on Reward Power). We expect positive correlations between the PDSS and the scale of self-efficacy because they are similar constructs. In both cases, individuals assess their ability to perform. Regarding the Leadership Based on Expert Power, we supposed a positive relationship with the PDSS because Professional development appears associated with transformational leadership style and not transactional (Mourão, 2018) and Leadership Based on Expert Power would be more associated with transformational leadership (Atwater and Yammarino, 1996). In contrast, we do not expect a significant correlation between PDSS and Leadership Based on Reward Power since this base is more associated with transactional leadership (Atwater and Yammarino, 1996). In fact, in transactional leadership, a contingent leader rewards followers for realizing agreed objectives and goals (Howell and Avolio, 1993).

On Criterion-Related Validity, despite limited previous work on this type of validity, we propose that the PDSS will demonstrate significant predictive ability, particularly for job performance (Shah et al., 2011; Borg, 2018) and job satisfaction (Borg, 2018; Hariyati and Safril, 2018). Therefore, we have two hypotheses: (1) Professional Development is positively correlated with the In-role performance and Job Satisfaction and (2) Professional Development is positively associated with career promotion. These hypotheses were tested in two ways: either using the Pearson correlation between Professional Development with In-role performance and Job Satisfaction, or using the *t*-test to compare means of professional development among those who had had a career promotion in the last 3 years and those who had not. For these analyses, we used the sample of Study 2 (Brazilian and Angolan). Values of *p* for each data set were calculated using an unpaired Student’s *t*-test with a 95% CI.

We followed established procedures for testing for method effects with latent variable modeling (Williams and Anderson, 1994). Briefly, these tests involve comparing the fit of the baseline model with that of a confounded measurement model (Ferris et al., 2008). For the baseline model, a given latent method effect variable (e.g., social desirability) is modeled as having no relation to the latent variable representing the PDSS. According to Ferris et al. (2008), the confounded measurement model differs from the baseline model in that it adds paths from the latent method effect variable to the indicators of the PDSS latent variable (i.e., the method effect is allowed to “confound” measurement of the PDSS). A significant improvement in Chi-square from the baseline model to the confounded measurement model suggests the presence of method effects. The data are better represented by allowing the method effect of influencing the PDSS at the measurement (i.e., indicator) level. Should the confounded measurement model provide a significantly better fit than the baseline model, one can estimate the variance the latent method effect accounts for in the indicators of the PDSS by squaring the path estimates from the latent method effect variable to the indicators of the PDSS (Williams and Anderson, 1994). We estimated the baseline separately for each method effect (i.e., social desirability and other variables).

#### Materials and methods

##### Participants

As this third study involved a more extensive set of variables, the sample was divided into two. Participants from both samples responded to the scale of professional development, and other scales were divided between the two samples. This procedure was adopted to avoid fatigue in completing the process responses.

In the first sample, 203 Brazilian workers participated. Of them, 60.3% were female, and 79.8% had completed college. The age of the respondents ranged from 18 to 69 (*M* = 38.59, *SD* = 11.02). Working time ranged from 1 to 42 years (*M* = 15.57, *SD* = 10.41). In the second sample, 240 Brazilian employees completed the questionnaire. Of them, 47.1% were female, and 53.7% had completed college. The age of the respondents ranged from 19 to 60 (*M* = 33.42, *SD* = 9.96). Working time ranged from 1 to 44 years (*M* = 14.26, *SD* = 9.88).

##### Measures

###### Professional development short scale

The same scale described in Study 2.

###### Social desirability scale

The Social Desirability Scale—SDS appears suitable in cross-cultural settings, with a rating-scale format with 17 items (Stöber, 2001), in the true-false response format. Some examples of the items are: “I always admit my mistakes openly and face the potential negative consequences,” and “In traffic, I am always polite and considerate of others.” The original scale was translated into Portuguese following the same procedure for other scales used in this study (according to recommendations of Borsa et al., 2013). The Cronbach Alpha coefficient in this sample for the Social Desirability Scale was 0.74.

###### In-role performance

Items were adapted from Goodman and Svyantek (1999). The Portuguese version was then generated using the backward translation technique. More specifically, two bilingual persons with Portuguese backgrounds independently translated the items into Portuguese. The two Portuguese copies were then sent to another bilingual professional for review and translation into English. An English native speaker reviewed the back-translated scale to confirm its equivalence with the original. Revisions were made in the Portuguese translation based on comments from the final reviewer. Participants could indicate the extent to which they found each of the statement’s characteristics of themselves (0 = *not at all characteristic* to 6 = *totally characteristic*). Two examples are: “Demonstrates expertise in all job-related tasks” and “Fulfills all the requirements of the job.” The Cronbach α coefficient in this sample for the in-role performance was 0.92.

###### General self-efficacy scale

The eight items of the General Self-Efficacy Scale (Chen et al., 2001) were adopted. The Portuguese version followed the same translation procedures for the In-role Performance Scale. The General Self-Efficacy Scale was scored on a five-point Likert-type scale from 1 (*strongly disagree*) to 5 (*strongly agree*). Two examples are: “I will be able to achieve most of the goals that I have set for myself” and “In general, I think that I can obtain outcomes that are important to me.” The Cronbach Alpha coefficient in this sample for the General Self-Efficacy Scale was 0.80.

###### Job self-efficacy

Based on preliminary focus group interviews, Chen et al. (2004) developed an eight-item Job Self-Efficacy Scale. The scale was scored on a seven-point Likert-type scale from 1 (*strongly disagree*) to 7 (*strongly agree*). Items from this scale included “I can effectively handle difficult tasks at work” and “I am able to solve most work problems in a timely fashion.” The Cronbach α coefficient in this sample for the General Self-Efficacy Scale was 0.85.

###### Job satisfaction

Job satisfaction was assessed using a scale similar to the Diagnostic Survey General Satisfaction Scale (Hackman and Oldham, 1975), each assessed by five items on a seven-point response scale: 1 (*strongly disagree*) to 7 (*strongly agree*). The job satisfaction scale consisted of items such as: “I am satisfied with my current job” and “My current job corresponds to what I always wanted.” The original scale was translated into Portuguese, following the same procedure for other scales. The Cronbach α coefficient in this sample for the job satisfaction scale was 0.93.

###### Career promotion

Career Promotion was measured using one question “Did you receive a promotion in the last 3 years?” Workers who responded “yes” to this question were defined as having recent career promotion. This question was used only to identify the PDSS to distinguish people who have achieved career advancement.

###### Bases of power

The survey included eight items (four items by dimension Reward and four items by dimension Expert) measuring followers’ perceptions of their supervisor’s bases of power (Yukl and Falbe, 1991). In the original study, seven power bases were assessed (36 items): Legitimate, Reward, Coercive, Expert, Referent, Persuasive, and Charismatic. In this study, only Reward and Expert were reported because of our hypotheses. The response format for the items ranged from 1 (*not at all, never true*) to 7 (*to a very great extent, almost always true*). The Cronbach Alpha coefficient in this sample for reward and expert bases of power were, respectively, 0.76 and 0.70.

#### Results

In this study, we tested the convergent, discriminant, and criterion validity of the PDSS. The results confirm the hypotheses of convergent validity because they show a pattern of positive correlations between the PDSS and some “criterion” variables. As expected, the PDSS correlated positively with the General Self-Efficacy Scale (*r* = 0.42, *p* < 0.01) and Job Self-Efficacy Scale (*r* = 0.50, *p* < 0.01), a moderate correlation and a strong correlation, respectively (according to the classification proposed by Hair et al., 2019). Results also showed evidence that professional development is not redundant with general self-efficacy and job self-efficacy. Indeed, none of the correlations between professional development and general or job self-efficacy approached a level to suggest multicollinearity (Lindner et al., 2020).

We also tested the relationship between the General Self-Efficacy Scale and Job Self-Efficacy Scale with the PDSS from ESEM analyses to confirm these results. In both cases, we compared a free estimation model and a model with coefficient 1 for the relationship between the PDSS and Self-Efficacy (general or on the job). As shown in Table 4, the unconstrained General Self-Efficacy and Professional Development models got a better fit index than the constrained model. The same can be observed for models of Job Self-Efficacy because the unconstrained model got a better fit index. Thus, the results confirm the hypothesis of convergent validity of the PDSS.

**Table 4:** The goodness of fit statistics for tests of Self-Efficacy and professional development.

		*χ* ^2^	Δ*χ*^2^	*df*	CFI	TLI	RMSEA	RMSEA (90%)	SRMR
*General Self-Efficacy*	Unconstrained model	200.7	—	53	0.832	0.790	0.125	0.107–0.144	0.097
	Constrained model	234.7	4.7	54	0.794	0.748	0.137	0.119–0.155	0.148
*Job Self-Efficacy*	Unconstrained model	200.0	—	53	0.841	0.823	0.125	0.107–0.143	0.087
	Constrained model	222.3	2.3	54	0.837	0.800	0.132	0.115–0.151	0.128

Furthermore, the relationship between professional development and leaders’ leadership style helped test the convergent validity (Leadership Based on Expert Power) and divergent validity (Leadership Based on Reward Power) of the PDSS. The initial hypothesis was confirmed because, on the one hand, Leadership Based on Expert Power had a positive and significant relationship with the PDSS (*r* = 0.34, *p* < 0.01). On the other hand, Leadership Based on Reward Power had a significant relationship with the PDSS showing a weak Pearson Correlation coefficient (*r* = 0.16, *p* < 0.01). The Exploratory Structural Equation Modeling (ESEM) analyses did not confirm these results. The model testing Leadership Based on Reward Power and Leadership Based on Expert Power as predictors of Professional Development shows that only Leadership Based on Expert Power presents significant estimates (0.33, *p* < 0.01), while Leadership Based on Reward Power shows no significant relationship (0.062, *p* = 0.34). The practical fit indices (SRMR = 0.057, RMSEA = 0.073, CFI = 0.921, TLI = 0.890) all indicated an acceptable fit for this model. Therefore, our initial hypotheses about convergent and divergent validity are supported.

In relation to criterion validity, we confirmed our hypotheses that there was a positive relationship between Professional Development with both In-role performance (*r* = 0.49, *p* < 0.01) and Job Satisfaction (*r* = 0.40, *p* < 0.01). According to Cohen (1992), there is a medium size effect in these Pearson coefficients; however, to better assess this effect size, it is necessary to compare these results with other studies (Funder and Ozer, 2019). Research carried out by Carvalho and Mourão (2021), for example, found a smaller effect between professional development and career adaptability (*r* = 0.32, *p* < 0.01) and a larger effect between professional development and the perception of employability (*r* = 0.66, *p* < 0.01). In this sense, in a comparative way, the effect sizes obtained in the present study can be classified as “medium.” Regarding career promotion, we also confirmed our hypothesis because *t*-tests were significant for the Brazilian sample [*t*(648) = −2.725, *p* < 0.01, Cohen’s *d* = 0.21] and the Angolan sample [*t*(621) = −5.414, *p* < 0.01, Cohen’s *d* = 0.44]. It should be noted that the Angolan sample violated the assumption of the equality of variance. Thus, the value presented by the JASP software already considers Levene’s test. It is a particularly important finding because it allows identifying a result of the PDSS on a variable that is not perceptual. The nomological network of the constructs measured by the PDSS was studied by examining its correlation with a variable identifying one of its possible consequences—professional career advancement.

With regard to the Method Effects, the PDSS was uncorrelated with social desirability (*r* = −0.05, *p* = 0.53) and correlated significantly with job satisfaction (*r* = 0.40, *p* < 0.01). The PDSS was uncorrelated with social desirability and correlated significantly with job satisfaction. In addition to the correlation results, there was no significant Chi-square change between the baseline model and the social desirability—confounded measurement model, ∆χ^2^(_1, N_ = 203) 1.0, *p* > 0.05, which indicated that social desirability did not influence the measurement of the PDSS. However, there were significant Chi-square changes between the baseline model and the job-satisfaction-confounded-measurement model, ∆χ^2^(_1, N_ = 203) 24.8, *p* < 0.05. These results suggest that job satisfaction influences the measurement of the PDSS. We next examined the magnitude of the influence of job satisfaction method bias by squaring the paths from the method factor to the PDSS indicators. The results indicated that job satisfaction accounted for less than 4% of the systematic variance. This effect is small. These results are consistent with those of other investigations of method effects (e.g., Williams and Anderson, 1994; Ferris et al., 2008), which have concluded that although method effects may exist, the size of the effects is essentially negligible. These results indicate that the effects of the method do not generate undue confusion on the scale.

In summary, the results of the third study confirm the Convergent, Discriminant, and Criterion Validity of the Professional Development Short Scale, and allow for discarding the bias of social disability on the perception of professional development. Therefore, we confirmed the hypotheses of Discriminant Validity with the General Self-Efficacy and Job Self-Efficacy Scales, and Convergent Validity (related to Leadership Based on Expert Power and not related to Leadership Based on Reward Power). These results are consistent with the literature revisited (Howell and Avolio, 1993; Atwater and Yammarino, 1996; Mourão, 2018).

We also confirmed the hypotheses about Criterion-Related Validity, as Professional Development is positively associated with In-role performance, Job Satisfaction, and Career Promotion (Borg, 2018; Hariyati and Safril, 2018). Finally, the procedures for testing for method effects with latent variable modeling (Williams and Anderson, 1994), comparing the fit of the baseline model with that of a confounded measurement model (Ferris et al., 2008) indicated that social desirability does not produce response bias in professional development perception.

### Study 4: Temporal stability

Four weeks later, a subset of the third sample (*N* = 203) participated in a second survey with the same questionnaires. Data analysis on test–retest reliability was carried out with 179 (88.2%) respondents. The demographic characters were like the third sample. To test the temporal stability, firstly, we used test–retest Pearson correlations. As the test–retest correlations could not detect any systematic increase or decrease in scores over time, we performed paired *t*-tests as another temporal stability test. Finally, using the repeated measures, we compared the Alpha de Cronbach in the test–retest reliability for the PDSS.

#### Materials and methods

##### Participants

A total of 179 Brazilian workers participated in the fourth study. Of them, 62.0% were female, and 78.8% had completed college. The age of the respondents ranged from 18 to 69 (*M* = 38.27, *SD* = 11.13). Working time ranged from 1 to 42 years (*M* = 15.55, *SD* = 10.50).

##### Measures

###### Professional development short scale

The same scale is described in Study 2 and Study 3.

#### Results

Test–retest correlations indicated acceptable temporal stability (*r* = 0.69, *p* < 0.01). The paired *t*-tests were conducted as another test of temporal stability. These *t*-tests were not significant for the PDSS [*t*(178) = −0.439, *p* = 0.661]. Test–retest reliability for the scale over the 4-week time period pointed out Alpha de Cronbach, respectively 0.78 and 0.81. The fit statistics for these two models were good (χ^2^ = 0.29, *df* = 2.00, *p* = 0.086, GFI = 0.999, CFI = 1.000, SRMR = 0.020, and RMSEA = 0.000 and χ^2^ = 0.07, *df* = 2.00, *p* = 0.096, GFI = 1.000, CFI = 1.000, SRMR = 0.011, and RMSEA = 0.000, respectively).

## Conclusion

The primary goal of this research is to present evidence of the validity and accuracy of Short Scale Professional Development for different occupational categories. Such a goal had been pursued by means of a comprehensive series of stages assembled in the studies reported in this paper.

Psychology researchers, in general, face an unavoidable dilemma between psychometric purity and practicality in the application of instruments. In addition, maintaining conciseness can be a challenge in screening studies, in large-scale surveys, in surveys that involve more variables, or in repeated measures experiments. In these different situations, short scales can be an advantageous solution (Romero et al., 2012).

Research evidence on the validity of the Professional Development Short Scale can provide researchers with a useful tool to diagnose the perception that employees have their own professional development. Besides its use in work organizations, the PDSS can as well be applied in order to evaluate and compare workers from different organizations in the same sector of activity. It may also be useful in guiding public policy training. Finally, the measure can also be helpful for career guidance and career planning.

We followed a strategy that allowed us to focus on fewer specific goals at each step of the way, building it incrementally. As a conclusion, the results pointed out in this article show that the psychometric properties of the PDSS are presumably suitable for application in extensive studies of professional development. Moreover, this research demonstrated the validity of the PDSS as regards its convergence-discriminant pattern with the General Self-Efficacy and Job Self-Efficacy Scales, and in its relationship to other relevant constructs (Bases of Power/leadership styles, In-role performance, Job Satisfaction, and Career Promotion). In all these cases, the results supported the initial hypotheses, as they are in conformity with the literature in the area (Howell and Avolio, 1993; Atwater and Yammarino, 1996; Mourão, 2018), job satisfaction (Borg, 2018; Hariyati and Safril, 2018), or in-role performance (Shah et al., 2011).

**Figure 2:**
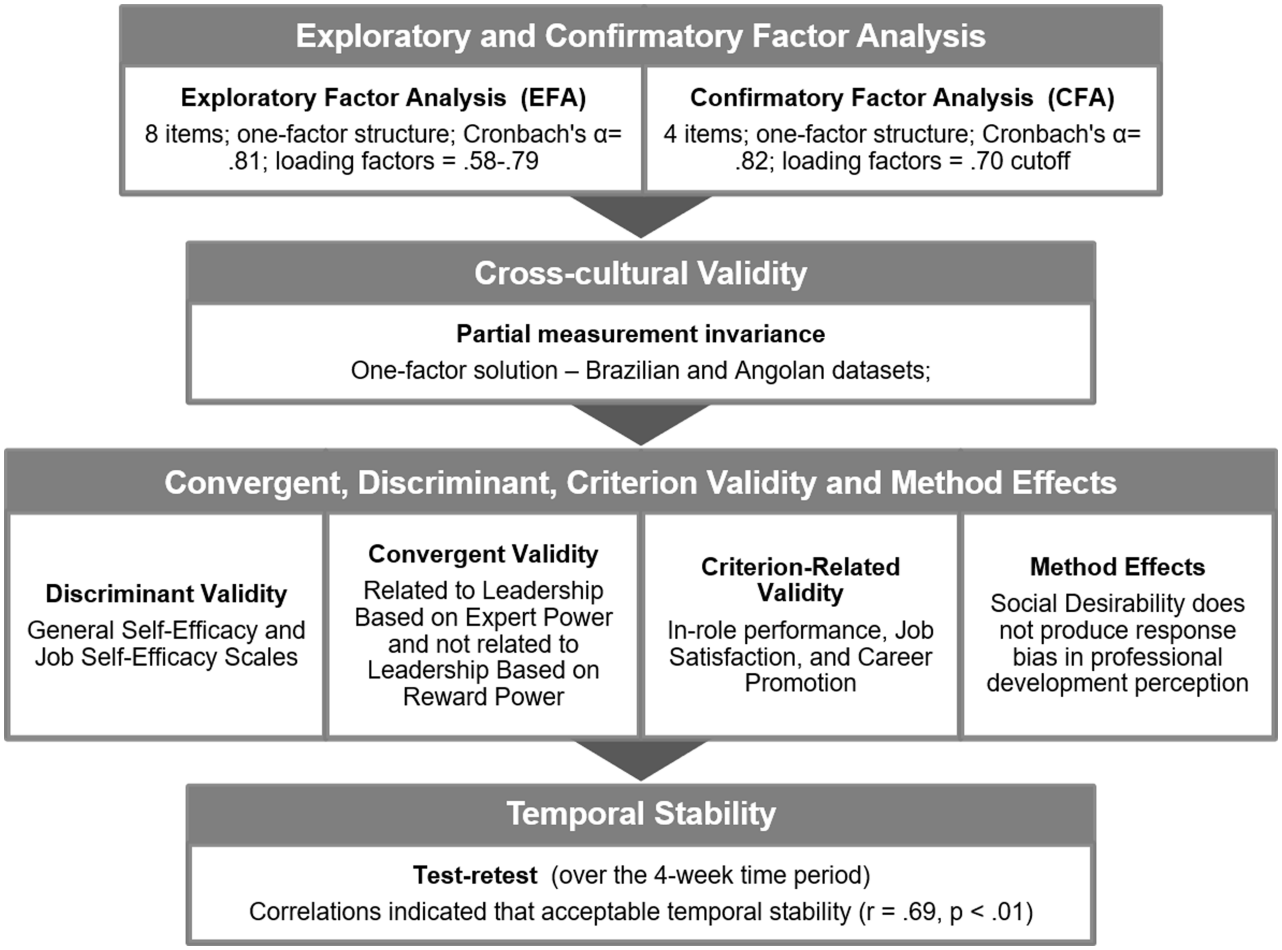
Evidence of the validity and accuracy of the Professional Development Short Scale (PDSS).

Furthermore, this study showed that the perception of professional development has no significant correlation with the Social Desirability Scale, which is vital given the limitation of the common method variance. The criterion-related validity analyses provided a coherent results profile with the concept of Professional Development (Fernandes et al., 2019; Figure 2).

Another important advantage that makes the PDSS highly applicable is the fact it is so concise. Since current studies try to integrate several variables, instruments need to prevent tedious response experiences through a collection of brief scales. The PDSS meets these criteria and therefore can be used integrated (Romero et al., 2012).

The current study has some limitations that may be the target of future research. First, the studies were completed only in Brazil and Angola. The study of the PDSS in other socio-cultural contexts is essential, though we expect the observed effects to generalize. Second, as with many studies in Industrial and Organizational Psychology, the measurement method (self-reporting for both predictor and criterion variables) may be contaminating the correlations. In this study, all variables but career promotion were of perceptual nature. Despite a 4-week interval between data collection of predictors variables and criteria, the common method bias is a concern. As such, in further studies, the use of different measurement methods and different statistical ways of identifying and correcting the effects of common method variance is highly recommended (Richardson et al., 2009). Studies explicitly intended to examine these effects are needed in order to more accurately assess the validity of short scales, and in line with the recommendations of Romero et al. (2012), studies with the explicit aim of examining such effects are proposed.

Despite these limitations and the need for greater refinement, the results of this study support the potential of the PDSS as an efficient measuring instrument for Professional Development. Of course, a short scale is unwarranted when specific aspects of Professional Development are being examined or when research projects have the time and resources to adopt longer scales (Schweizer, 2011; Romero et al., 2012). However, short scales can help the scientific community examine Professional Development in multiple areas of psychological and organizational research. Moreover, as it is a widely applicable instrument, there is potential for use in large-scale cross-cultural studies. This does not mean that researchers should make indiscriminate use of short scales, nor that the refinement of the professional development scale can be disregarded. However, for surveys that require brevity, the potential benefits of short scales, such as the PDSS, may be worth it.

## Data availability statement

The raw data supporting the conclusions of this article will be made available by the authors, without undue reservation.

## Ethics statement

The studies involving human participants were reviewed and approved by Comitê de Ética em Pesquisa em Humanos e Animais da Universidade Salgado de Oliveira (CEP-UNIVERSO). The patients/participants provided their written informed consent to participate in this study.

## Author contributions

LM and ST contributed to the conception and design of the study. LM organized the database, performed the statistical analysis, and wrote the first draft of the manuscript. LM, ST, and HS wrote sections of the manuscript. LM and HS contributed to manuscript revision, read, and approved the submitted version. All authors contributed to the article and approved the submitted version.

## Funding

This work was supported by Coordenação de Aperfeiçoamento de Pessoal de Nível Superior (Code 001); Fundação de Amparo à Pesquisa do Estado do Rio de Janeiro (E-26/202.913/2018); Conselho Nacional de Desenvolvimento Científico e Tecnológico (311162/2021-5); and Fundação para a Ciência e a Tecnologia (UIDB/00315/2020).

## Conflict of interest

The authors declare that the research was conducted in the absence of any commercial or financial relationships that could be construed as a potential conflict of interest.

## Publisher’s note

All claims expressed in this article are solely those of the authors and do not necessarily represent those of their affiliated organizations, or those of the publisher, the editors and the reviewers. Any product that may be evaluated in this article, or claim that may be made by its manufacturer, is not guaranteed or endorsed by the publisher.
